# Unusual histopathological diagnosis of prostatic blue nevus: a case report

**DOI:** 10.1186/1752-1947-7-291

**Published:** 2013-12-30

**Authors:** Nelson Montalvo, Ligia Redrobán

**Affiliations:** 1 Servicio de Patología, Hospital Metropolitano, Av. Mariana de Jesus s/n y Nicolás Arteta, Quito, Ecuador

**Keywords:** Benign prostatic hyperplasia, Melanocytic lesion, Prostatic blue nevus

## Abstract

**Introduction:**

Prostatic blue nevus was first described as a benign lesion of uncertain and controversial histogenesis by Nogogosyan in 1963. Currently, 30 cases have been reported in the world literature.

**Case presentation:**

A 63-year-old Hispanic man presented with prostatism of several months’ evolution. Histopathological examination revealed a blue nevus associated with nodular hyperplasia and acute inflammation.

**Conclusion:**

Prostatic blue nevus is a rare and unusual, histologically benign prostatic lesion with limited clinical significance and a favorable prognosis.

## Introduction

Melanocytic lesions, comprising blue nevi (BN), melanosis and malignant melanomas, are neoplastic processes observed, though rarely, in the prostate gland [1]. Extracutaneous location of a BN is extremely unusual. There have been reports of BN in the spermatic cord, prostate, vagina, cervix, oral cavity, paranasal sinuses, larynx, breast, pulmonary hilum and lymph nodes [2-4].

Prostatic blue nevus (PBN), first described in 1963 by Nogogosyan, is a benign pigmented lesion similar to its cutaneous counterpart, in which melanin pigment is deposited in the cytoplasm of normal, hyperplastic or malignant stromal cells. Its identification is usually incidental, after a histopathological study. Its pathogenesis remains unclear and controversial, but the accepted hypothesis is that of the centrifugal migration of melanoblasts from the neural crest to the prostate during the embryonic period, resulting in their ectopic location in this organ and in others. Currently, 30 cases have been reported in the world literature [5,6].

## Case presentation

We present the case of a 63-year-old Hispanic man with a history of type 2 diabetes mellitus, dyslipidemias and right nephrectomy due to a tumor 8 years previously, who presented with prostatism with a 3-month progress. He was diagnosed with benign prostatic hyperplasia during a urological examination. A digital rectal examination revealed an enlarged gland with a soft nodule in the left lobe; prostate specific antigen was within normal limits (3.4ng/mL) and a suprapubic prostatectomy was performed.

**Figure 1:**
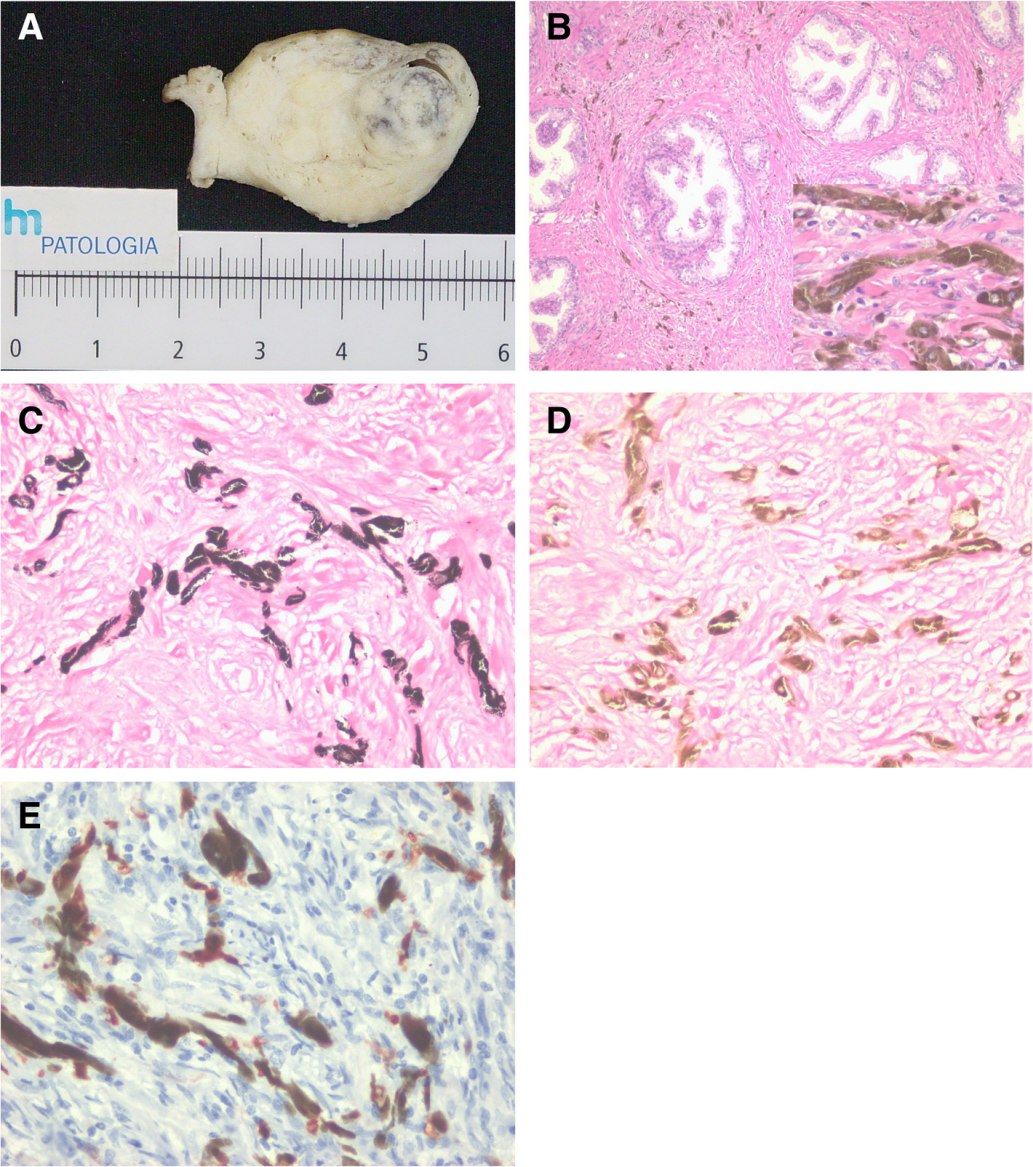
**Prostatic blue nevus. A)** Macroscopic study, prostate gland, left lobe with a well-defined 1.4cm blackish area. **B)** Dusty and granular melanin pigment within myofibroblasts in the stroma (hematoxylin and eosin stain; inset ×40). **C)** Masson-Fontana stain highlights pigmented stromal cells, confirming the presence of melanin (×40). **D)** Negative iron reaction in the pigmented stroma cells (×40). **E)** Pigmented stroma cells immunopositive for S100 protein (×40).

Macroscopic study showed a surgical specimen measuring 4.5×4×2cm, weighing 27g that, when cut open, was multinodular, elastic in consistency, with a well-defined blackish area 1.4cm in diameter in the left lobe (Figure 1A). Microscopy revealed nodular hyperplasia associated with an acute inflammatory process and a blue nevus comprising fusiform stromal cells with intense melanin pigment (Figure 1B). Masson-Fontana (+) and iron (−) histochemical techniques and S-100 (+) immunohistochemistry supported the diagnosis (Figure 1C-E).

## Discussion

Melanin is an endogenous pigment derived from tyrosine and present in several normal human tissues such as the retina, iris, skin and central nervous system, as well as in benign lesions (nevi) and malignant lesions (melanomas). The origin of ectopic melanocytes remains controversial. It has been postulated that melanoblasts migrate from the neural crest along the mesoderm, differentiating into melanocytes in the stroma of various organs and tissues.

Blue nevus of the prostate is histologically similar to its cutaneous counterpart; it is characterized by a proliferation of spindle-like stromal cells with abundant intracytoplasmic melanin pigment [1,2]. The differential diagnosis of PBN should include prostatic pseudomelanosis or lipofuscinosis in which the cytoplasm of prostatic epithelial cells contains brownish or hematoxylinic Masson-Fontana and iron-negative lipofuscin granules. These pigmentations are much more frequent, increase considerably with cellular senescence or aggression, and generally go unnoticed by microscopic examination. Other pigments, such as hemosiderin (Fe +) secondary to hemorrhage or prostate stroke or, less frequently, to hemochromatosis, must be taken into consideration [3,4].

PBN is a rare lesion exclusively diagnosed incidentally, especially after histopathological examination of simple suprapubic prostatectomy specimens, as occurred in our case. The literature shows that case reports of PBN mostly concern suprapubic prostatectomy specimens and transurethral resection is commonly involved, whereas only two fine-needle biopsy cases have been reported [1-3].

It is vital to exclude a diagnosis of primary or metastatic malignant melanoma of the prostate, which constitutes less than 1% of all melanomas. Such a diagnosis is difficult and results in aggressive surgical treatment. Very few cases of melanoma have been published and most have been secondary, either metastatic or arising in the prostatic urethra. Given the negligible possibility of malignant transformation, additional periodic checks are not advisable after a PBN diagnosis [5,6].

## Conclusion

We report the case of a PBN incidentally associated with nodular hyperplasia and superimposed acute inflammation. PBN is a rare and unusual, histologically benign prostatic lesion with limited clinical significance and a favorable prognosis.

## Consent

Written informed consent was obtained from the patient for publication of this case report and accompanying images. A copy of the written consent is available for review by the Editor-in-Chief of this journal.

## Competing interests

The authors declared that they have no competing interests.

## Authors’ contributions

NM performed the histological examination and diagnosis; LR conducted a thorough literature review and manuscript preparation. The authors read and approved the final manuscript.
